# Supplementary material for: Fabrication of PbO2 Electrodes with Different Doses of Er Doping for Sulfonamides Degradation
Source: Int J Environ Res Public Health. 2022 Oct 19;19(20):13503. doi: 10.3390/ijerph192013503 (PMC9602837; doi:10.3390/ijerph192013503)
Supplement: Supplementary file 1 [file ijerph-19-13503-s001.zip › ijerph-1906685-supplementary.pdf]

Supplementary Materials

Fabrication of PbO_2 Electrodes with Different Doses of Er Doping for Sulfonamides Degradation

Tianyu Zheng, Chunli Wei, Hanzhi Chen, Jin Xu, Yanhong Wu*, Xuan Xing*

Department of Environmental Science, College of Life and Environmental Science, Minzu University of China, Beijing 100081, China

Number of Pages (including this cover sheet): 10

Number of Tables: 3

Number of Figures: 4

* Corresponding author. Tel.: +86 10 68933621.

E-mail: xingxuanpku@163.com. (X. Xing)

Table S1. Accelerate life time of prepared electrodes.

	$Ti/Sb\text{-}SnO_2/PbO_2$	TiO_2-NCs/Sb-SnO_2/PbO_2
Accelerate life time/h	10	35
Service lives/year	2.85	9.99

Accelerated lifetime was performed by anodic polarization of the different electrodes at 1 A cm^{-2} in a 0.2 M H_2SO_4 solution. The anode potential was measured as function of time and the electrode was considered to be deactivated when the potential increased to 10 V from its initial value. The lifetime practical application has been calculated according to Eq. (1):

$$t = (A_1/A)^2\ t_1 \qquad (1)$$

where A_1 is the current density in the accelerated test (1 A/cm^2), A is the current density in practical applications (20 mA cm^{-2}), t_1 is the lifetime of the electrode in the accelerated test (h), and t is the lifetime in practical applications (h).

Table S2 Percentage of different elements in Er-PbO_2 by EDS

	O		Pb		Er	
	Weight Percentage	Atomic Percentage	Weight Percentage	Atomic Percentage	Weight Percentage	Atomic Percentage
0% Er-PbO_2	12.23	64.34	87.77	35.66	0	0
0.5% Er-PbO_2	12.31	64.51	87.52	35.41	0.09	0.05
1% Er-PbO_2	12.28	64.45	87.57	35.48	0.14	0.07
2% Er-PbO_2	10.94	61.39	88.87	38.50	0.19	0.10
4% Er-PbO_2	11.81	63.40	87.85	36.42	0.34	0.18

Table S3 Parameters of the first-order reaction kinetics

Experiment condition		k (h^{-1})	R^2
Electrodes	0%PbO_2	0.93	0.97
	0.5%(Er-PbO_2)	1.19	0.99
	1.0%(Er-PbO_2)	1.22	0.97
	2.0%(Er-PbO_2)	1.39	0.99
	4.0%(Er-PbO_2)	1.25	0.99
Current Density	10 $mA\cdot cm^{-2}$	0.96	0.93
	20 $mA\cdot cm^{-2}$	1.33	0.96
	30 $mA\cdot cm^{-2}$	1.42	0.99
pH	3.0	1.59	0.97
	7.0	1.38	0.92
	11.0	0.63	0.98

Table S4 Electron density of each atom of SMR

atom	charge	atom	charge	atom	charge	atom	charge	atom	charge
1C	-0.119	8S	1.242	15C	-0.139	22H	0.084	29H	0.111
2C	-0.056	9O	-0.551	16C	-0.364	23H	0.265	30H	0.140
3C	-0.199	10O	-0.517	17N	-0.516	24H	0.266		
4C	-0.081	11N	-0.698	18N	-0.469	25H	0.307		
5C	-0.115	12C	0.653	19H	0.083	26H	0.106		
6C	0.299	13C	0.125	20H	0.137	27H	0.093		
7N	-0.656	14C	0.297	21H	0.129	28H	0.142		

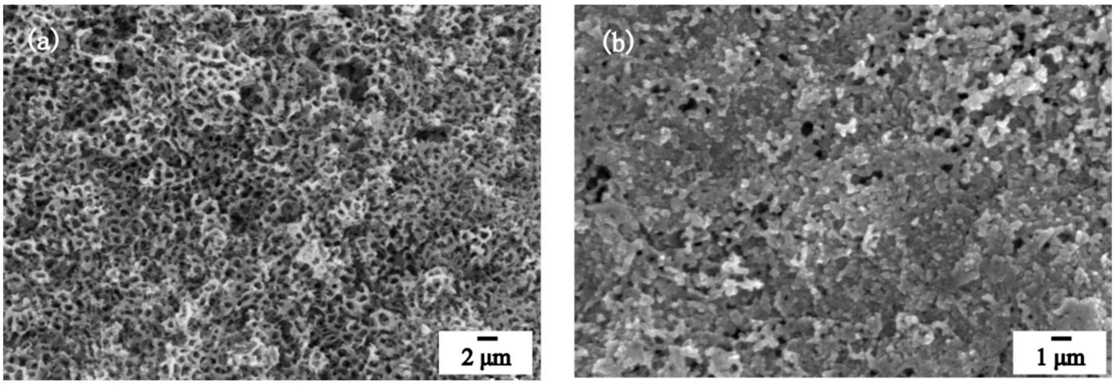

Figure S1 SEM of TiO_2-NCs and TiO_2-NCs/SnO_2-Sb

The electrode preparation process was as following:

Titanium sheets (20 mm × 15 mm × 1.5 mm) were polished by grinding cloths of 230, 400, and 600 meshes. Then, they were cleaned ultrasonically in ultrapure water, acetone, and ultrapure water for 15min each in sequence. Afterwards, the titanium sheets were immersed in boiling 18% HCl at the temperature of 85 °C for 10 min. Before electrodeposition, titanium sheets were treated by anodization in the electrolysis system with Pt electrode as counter electrode. The space between anode and cathode was set to be 1.5 cm. Electrolysis was carried out in electrolytes consisting of NaF (0.8 wt%), Na_2SO_4 (1.6 wt%), polyethylene glycol (PEG, 10 wt%), and ultrapure water (87.6 wt%) with a voltage of 30 V at room temperature. After 45 min of electrolysis with stirring, the nanocrystalline coral-like TiO_2 (TiO_2-NCs) substrate was obtained. Then, the TiO_2-NCs substrates were annealed in oxygen atmosphere at 500 °C for 1.5 h for crystallization and the rates for heating and cooling were set as 1 °C min^{-1} [9]. Ultrapure water from a Millipore Milli-Q system (>18 mΩ cm^{-1}) was used

for all solutions' preparation at 25 ± 1 °C.

The service lifetimes of Ti/SnO_2-Sb/PbO_2 and TiO_2-NCs/ SnO_2-Sb/PbO_2 were tested and compared. The accelerated lifetime was 10 h for Ti/SnO_2-Sb/PbO_2 while it was 25 h for TiO_2-NCs/ SnO_2-Sb/PbO_2. The service lives also have been calculated, which were 2.85 and 9.99 year for Ti/SnO_2-Sb/PbO_2 and TiO_2-NCs/ SnO_2-Sb/PbO_2, respectively. This phenomenon demonstrated that TiO_2-NCs prolonged the service life significantly.

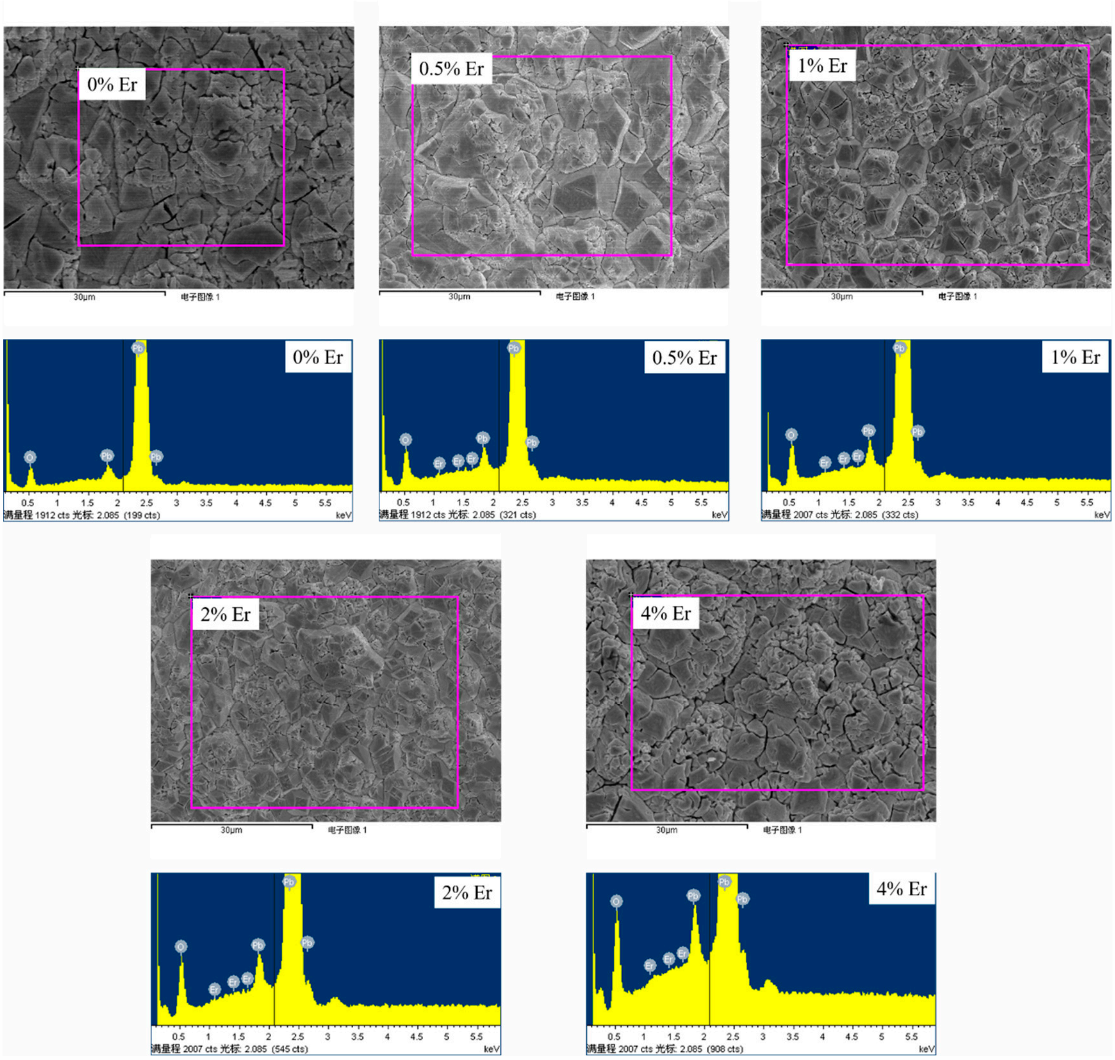

Figure S2 EDS results of different Er-PbO_2 electrodes

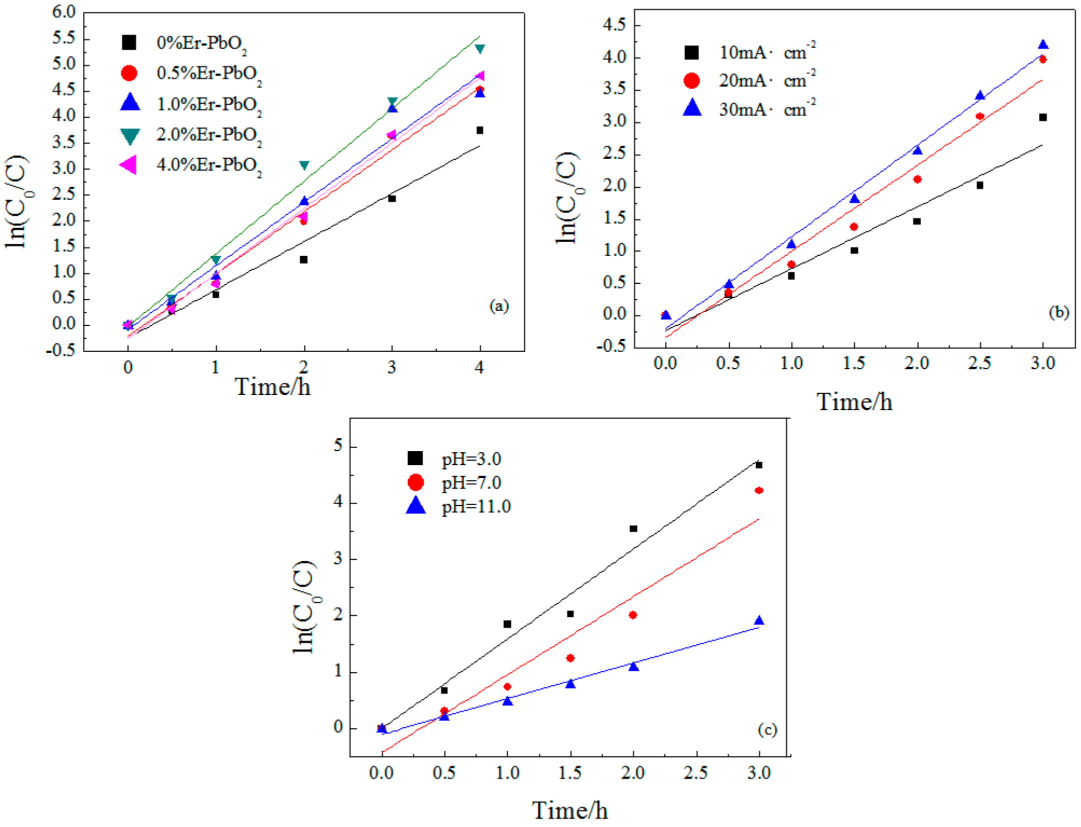

Figure S3. Kinetics analysis at different electrodes (a), current densities (b), pH(c) in electrochemical degradation process.

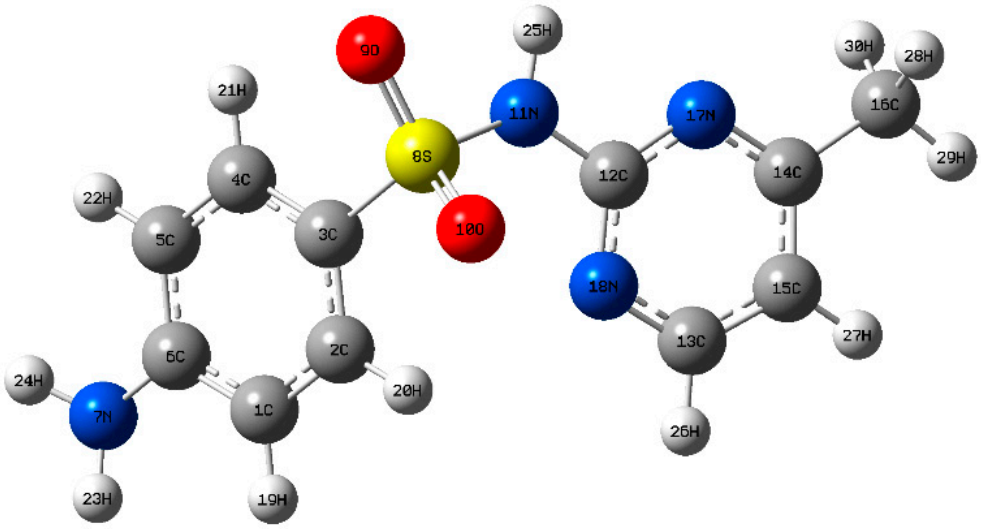

Figure S4. The optimized SMR configuration.

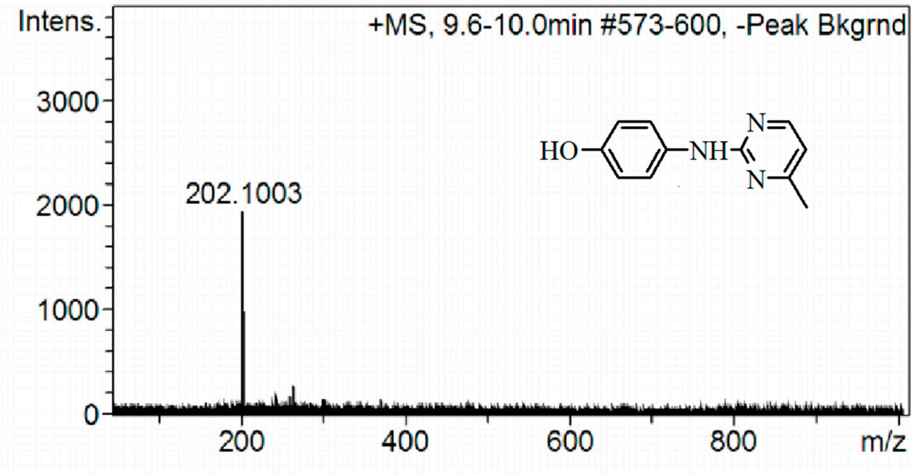
Intens.
+MS, 9.6-10.0min #573-600, -Peak Bkgrnd
3000
2000
1000
0
202.1003
HO
NH
N
N
200
400
600
800
m/z

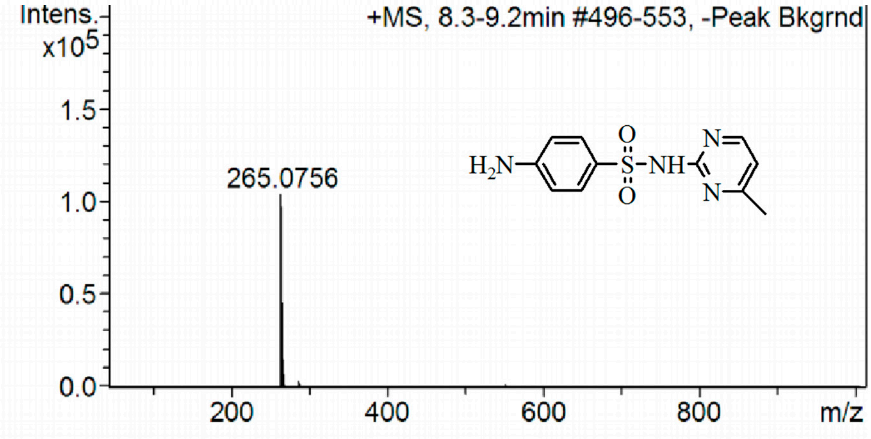
Intens.
x10^5
+MS, 8.3-9.2min #496-553, -Peak Bkgrnd
1.5
1.0
0.5
0.0
265.0756
H2N
O
S
O
NH
N
N
200
400
600
800
m/z

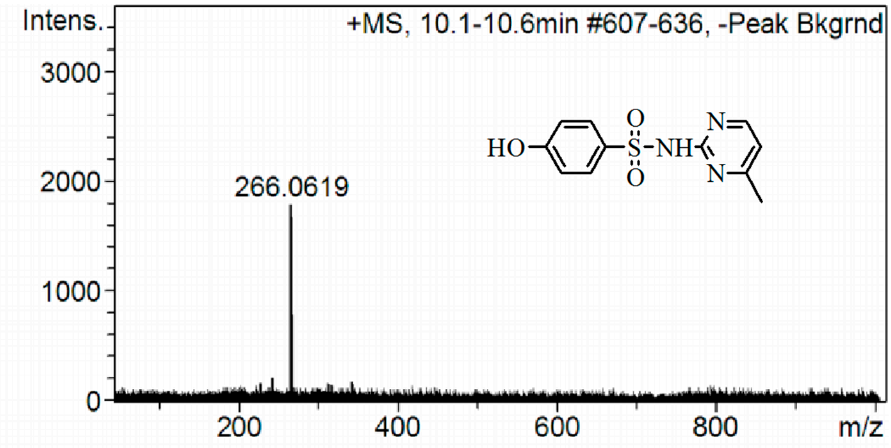
Intens.
+MS, 10.1-10.6min #607-636, -Peak Bkgrnd
3000
2000
1000
0
266.0619
HO
O
S
O
NH
N
N
200
400
600
800
m/z

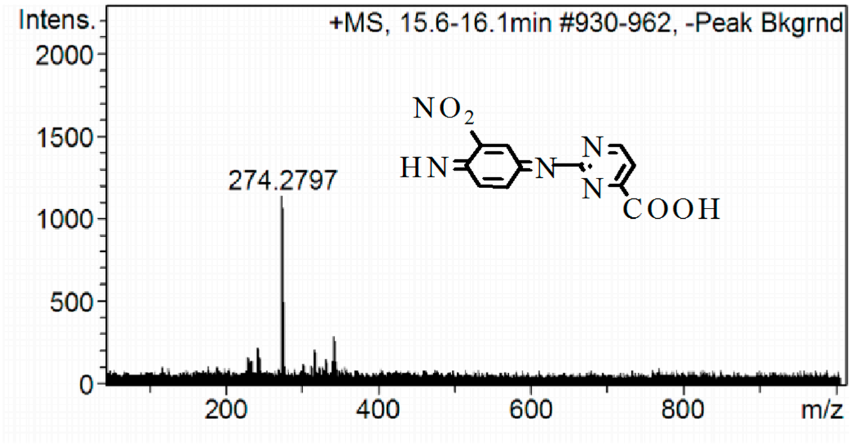
Intens.
+MS, 15.6-16.1min #930-962, -Peak Bkgrnd
2000
1500
1000
500
0
274.2797
NO2
HN
N
N
N
COOH
200
400
600
800
m/z

Figure S5 HPLC-Q-TOF-MS/MS images of intermediate products degraded under neutral conditions.
